# Effects of ethanolic extract of *Jasminum grandiflorum *Linn. flowers on wound healing in diabetic *Wistar *albino rats

**Published:** 2017

**Authors:** Hiren Hirapara, Vishal Ghori, Ashish Anovadiya, Seema Baxi, Chandrabhanu Tripathi

**Affiliations:** 1 *GMERS Medical College, Junagadh , Gujarat, India*; 2 *Billev Pharma East, Ljubljana, Slovenia*; 3 *Government Medical College, Bhavnagar, Gujarat, India*

**Keywords:** Jasminum grandiflorum Linn. Flowers, Streptozotocin-induced diabetes, Granulation tissue

## Abstract

**Objective:**

To evaluate wound healing activity of ethanolic extract of *Jasminum grandiflorum *Linn. (*J. grandiflorum*) flowers in diabetic rats.

**Materials and Methods:**

Streptozotocin-induced diabetic *Wistar* albino rats were divided into six groups (n=6).Three groups – diabetic control, positive control (that received Glibenclamide) and treatment (that received *J. grandiflorum *Linn. Flower extract) were operated for excision wounds (EW). These groups were evaluated for wound contraction and re-epithelization. The other three groups were operated for incision wounds (IW) and dead space wounds (DW). Incision and dead space wounds were produced in the same rats. IWs were analyzed for wound breaking strength and the granulation tissues from DWs were analyzed for dry weight, hydroxyproline content, and histology.

**Results:**

IWs and DWs showed significant improvement in wound breaking strength (265.8±10.4 vs 332.5±8.2; p<0.05), granulation tissue dry weight (26.1±0.6vs 40.4±0.3; p<0.01) and hydroxyproline content (19.3±0.5 vs 32.6±0.8; p<0.01) in treatment group as compared to control group. Neo-angiogenesis was also high in treatment group. Wound contraction was earlier (day 14) in treatment group compared to diabetic control (day 20). No significant improvement was seen in re-epithelization in treatment group.

**Conclusion:**

Ethanolic extract of *J. grandiflorum *Linn. flowers increases granulation tissue formation as well as neo-angiogenesis. It also enhances wound contraction; however, re-epithelization was not significantly affected. *J. grandiflorum *Linn. flowers could be potentially effective in promotion of diabetic wounds healing by increasing granulation tissue formation and enhancing wound contraction; however, further studies are required for its clinical application.

## Introduction

The wound healing is a highly dynamic and complex process that involves cellular, physiological and biochemical events, leading to re-establishment of structural integrity and functional restoration of injured tissue. Impaired wound healing in patients with diabetes mellitus (DM) imposes high morbidity and health care cost (Singer and Clark, 1999 ▶; Ramsey et al., 1999 ▶). It is one of the common complications associated with diabetes (Falanga, 2005 ▶). Diabetic patients often ignore lower extremity ulcers, which often, in their severe form, lead to amputation (Anderson, 2003 ▶). Causative mechanisms associated with impaired wound healing in diabetes are micro and macro vascular abnormalities, impaired epithelization and reduced angiogenesis (Traub and Bibber, 1995 ▶).

*Jasminum grandiflorum *Linn. (“*Spanish jasmine” *or *“Royal jasmine”*) is a plant of Oleaceae family. Its classical names are Jati, Malti or Rajputrika (Paarakh and Paarakh, 2009 ▶) and its flowers and leaves are widely used in folk medicine to prevent and treat breast cancers. Flowers are also useful in uterine bleeding when brewed as tonic (Mishra et al., 2010 ▶). *J. grandiflorum *Linn. contains triterpenoids, alkaloids, tannins, flavonoid, steroid, glycoside, terpenes, resins and salicylic acid (Nayak and Krishna, 2007 ▶; Patil and Saini, 2012 ▶). Moreover, flavonoids and triterpenoids are known to promote the wound-healing process due to their astringent and antimicrobial properties (Villegas et al., 1997 ▶; Tsuchiya et al., 1996 ▶). These compounds have antioxidant and antiulcer activities as well (Umamaheswari et al., 2007 ▶). 

Enzymatic wound debridement may be the primary technique in some cases when surgical debridement is not possible, to promote wound healing (Ramundo and Gray, 2008 ▶). Protease enzymes found from various sources like plants, microbes, maggots and animals have been found to be useful in wound debridement (Walsh (Ed), 2003 ▶). A previous study showed that the protease enzymes present in *J. grandiflorum *Linn. may be responsible for its wound healing property (Vidyalakshmi and Selvi, 2013 ▶). An ethanolic extract of *J. grandiflorum *Linn. flowers has been found to improve wound healing in the excision, incision and dead space wounds in non-diabetic rats (Nayak and Krishna, 2007 ▶). The present study was designed to evaluate the wound healing effects of ethanolic extract of *J. grandiflorum *Linn. flowers in diabetic rats.

## Materials and Methods

The study was started after obtaining approval from Institutional Animal Ethics Committee (IAEC), Government Medical College, Bhavnagar (GMCB), Gujarat (India) (Approval No. – 26/2012; Pharmacology No. – 24/2012). *Wistar*albino rats of either sex (weighing 200 – 350 g) were placed in individual polypropylene cages and acclimatized to laboratory environment for one week. The rats had free access to normal laboratory food and water *ad libitum*, and were kept under controlled room temperature (25±2 °C with 60–70 % humidity) and 12 hr-12 hr light-dark cycle.

**Streptozotocin (STZ) - induced diabetes**

A single dose of streptozotocin 50 mg / kg (Alfa Aesar, A Johnson Matthey Company, MA/USA,) was given intraperitoneally to induce diabetes mellitus (Ghori et al., 2014 ▶). After four days, random blood sugar (RBS) was measured from a blood drop collected from the tail vein, using glucometer (Accu – Chek Go, Roche Diagnostic, Germany). The rats showing RBS >300 mg/dl were included in the study. To maintain RBS between 250–350 mg/dl, Insulin Neutral Protamine Hagedorn (NPH) (5 IU/kg; Wosulin, Wockhardt Limited, Aurangabad, India) was given subcutaneously, once a day.

**Experimental design, and wound models**

Thirty six diabetic *Wistar *albino rats were divided into six groups (n=6 in each group). Three groups - Group I (Diabetic control), Group II (Glibenclamide –positive control) and Group III (*J. grandiflorum *Linn. flower- treated group) served as the excision wound groups and rats in these groups received distilled water 1 ml, glibenclamide (SIGMA Life science, New Delhi, India) 0.5 mg/kg and ethanolic extract of *J. grandiflorum *Linn. flower 250 mg/kg daily till complete healing of the excision wounds. Group IV (Diabetic control), Group V (Glibenclamide – positive control) and Group VI (*J. grandiflorum *Linn. flower) served as incision and dead space wound groups and received the same doses of respective treatment agents for 11 days as described above. Ethanolic extract of *J. grandiflorum *Linn. flower was procured from Leopard Investments Ltd., Gujarat, India. Glibenclamide and extracts were dissolved in distilled water and given orally. All the wounds were inflicted under ketamine (75 mg/kg) and xylazine (10 mg/kg)-induced anaesthesia. All aseptic precautions were taken during infliction of wounds.

**Excision wound**

A full-thickness circular skin (measuring approximately 500 mm^2^) section was excised from the nape of neck to produce excision wound (Morton and Malone, 1972 ▶).Wound contraction was evaluated by tracing wound margin on transparent plastic sheets. Wound area was measured soon after wounding and on days 3, 7 and 11. Re-epithelization was measured on the 11^th^ day of wounding when epithelization was visible. The plastic sheet was scanned and the wound area was measured using a UTHSCA image analyzer (version 3.00, The University of Texas Health Science Center, San Antanio, USA).Wound closure rate was calculated using the following formula (Ghori et al., 2014 ▶): 

Wound closure rate (%) = (Area_day 0_−Area_day n_ / Area_day 0_) × 100

Where Area_day 0 _=initial wound area on day 0

Area_day n _=area on the n^th ^post-wounding day

The wound re-epithelialization was calculated using the formula mentioned below (Ghori et al., 2014 ▶):

Re−epithelialization (%) on the 11^th^day = Total wound area (%) −Wound area not covered with epidermis (%).

**Re-sutured incision and dead space wound**

Full-thickness para-vertebral incisions of 5-cm length were made on either side of the vertebral column and sutured with black silk 4.0 sutures (Ehrlich and Hunt, 1969 ▶). Dead space wounds were produced in the same rats by inserting and suturing sterile grass pith (2.5 cm×0.3 cm) in the loose areolar tissue of the groins on either side. A sterile cotton pellet (weighing 10 mg) was inserted and sutured in loose areolar tissue of axillary region of either side. The sutures were removed on the 7^th^ day of wounding in incision wounds and on the 11^th^ day in dead space wounds. The incision wound breaking strength was measured on the 11^th^ post-wounding day by constant water flow technique described by Lee (Lee, 1968 ▶) under anaesthesia. The rats were sacrificed with high dose of ketamine and xylazine after measuring wound breaking strength. The granulation tissue formed on grass piths was utilized for the hydroxyproline estimation (Woessner, 1961 ▶) and histological examination. The hydroxyproline content was expressed as μg/100 mg of granulation tissue. The cotton pellets were excised from axillary region and dried overnight at 60^o^C in hot air oven. The weight of dried cotton pallet along with granulation tissue was expressed as mg / 100 g body weight (Dipasquale and Meli, 1965 ▶).

Haematoxylin and Eosine (H & E)-stained sections of granulation tissues were semi-quantitatively analyzed by a pathologist and given a grade of 1 – 4 for the presence of polymorphonuclear cells, macrophages, fibroblasts and neo-angiogenesis (Abramov et al., 2007 ▶).

**Statistical analysis**

All statistical analyses were done by using Graphpad instat demo version 3.0. The data were expressed as mean±Standard Error of Mean (SEM). One way analysis of variance (ANOVA) followed by Tukey-Kramer test for parametric variables and Kruskal-Wallis followed by Dunn’s multiple comparison test for non-parametric variables were used to compare differences among means of different groups. Statistically significant differences were considered if p<0.05.

## Results

**Excision wound**

**Figure 1:**
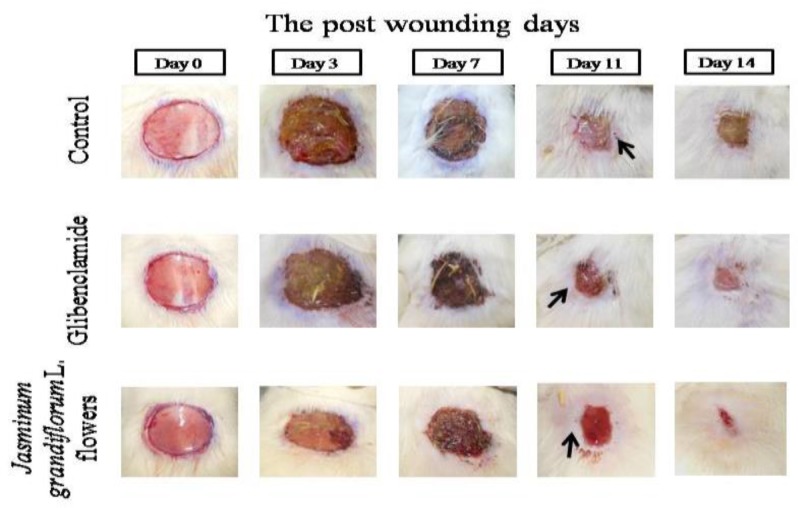
The excision wound healing time course noted on post-wounding days 0, 3, 7, 11 and 14

**Figure 2:**
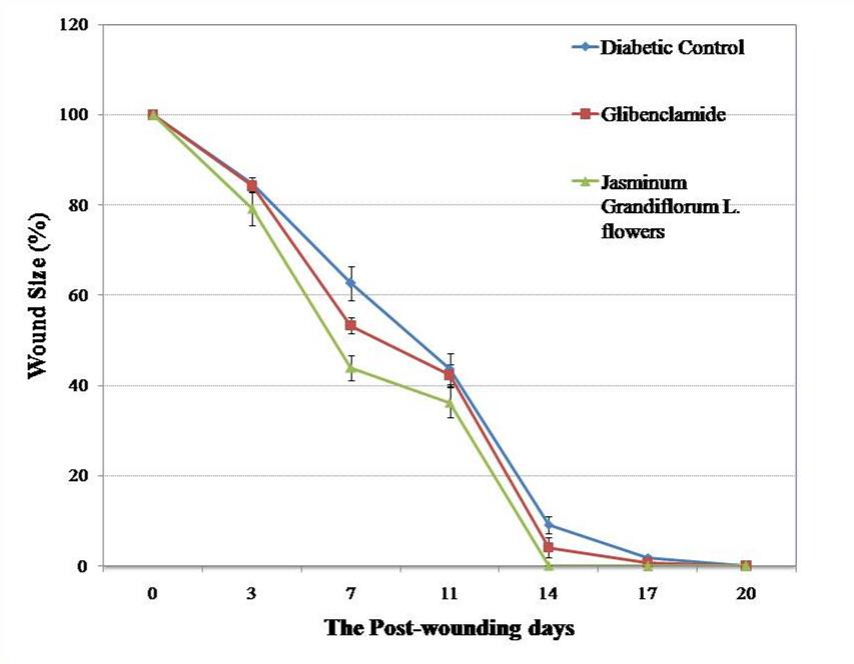
The wound healing curve shows the percentage of excision wound closure over the time period

The wound closure in all the groups is shown in Figure 1. Wound contraction was significantly higher in treatment group than diabetic control group on days 7 and 14 but it could not reach a statistically significant difference on day 11 (Table 1). Re-epithelialization on the 11^th^ post-wounding day was non-significantly higher than diabetic and positive control (Table 1). Initially, the wound closure was rapid (owing to wound contraction) followed by relatively slow healing as seen in Figure 2. Complete healing of wound was observed on the 14^th^ day in treatment group, on the 17^th^ day in positive control and on the 20^th^ day in diabetic control. There was no significant difference in re-epithelialization between the treatment and positive as well as diabetic control group.

**Re-sutured incision and dead space wound**

Mean incision wound breaking strength on the 11^th^ post-wounding day and granulation tissue dry weight in treatment group were significantly higher as compared to diabetic control group (Table 2). There was no significant difference in these parameters between treatment and positive control (glibenclamide) group. The hydroxyproline content in granulation tissue in treatment group was significantly higher than both the diabetic control and glibenclamide group (Table 2).

A semi-quantitative histological analysis of granulation tissue obtained on 11^th^ post-wounding day did not show any difference in any of the parameters examined except for the neo-angiogenesis, which was higher in treatment group compared to diabetic as well as positive control group (Table 3).

**Table 1:** Effect of ethanolic extract of *Jasminum grandiflorum* Linn. flowers on excision wound model

**Groups** **(n=6)**	**Wound closure (%)**					**Re-epithelialization** **(%) 11** ^th^ ** Day**
	3^rd^ Day	7^th^ Day	11^th^ Day	14^th^ Day	17^th^ Day	
**Diabetic control**	15.3±1.4	37.3±3.8	56.3±3.4	90.7±1.9	98.2±0.5	56.01±2.5
**Glibenclamide**	15.7±1.2	46.7±1.8	57.6±2.5	95.9±2.2	99.3±0.4	63.2±1.8
***Jasminum grandiflorum*** ** Linn. flowers**	20.8±3.6	56.0±2.8*	63.7±3.3	99.9±0.01*	100±0.0	64.5±3.5

n=6 in each group. Values are represented as mean±SEM;

* p<0.05 as compared to diabetic control (Tukey Kramer multiple comparison test).

**Table 2:** Effect of ethanolic extract of *Jasminum grandiflorum* Linn. flowers on incision and dead space wound model.

**Group**	**Incision wound breaking strength (g)**	**Granulation tissue dry weight ** **(mg/100 g body weight)**	**Hydroxyproline (µg/100 mg granulation tissue)**
**Diabetic control**	265.8±10.4	26.1±0.6	19.3±0.5
**Glibenclamide**	369.2±8.9*	37.5±0.9*	24.4±0.9
***Jasminum grandiflorum *** **Linn.** **flowers**	332.5±8.2*	40.4±0.3*	32.6±0.8*#

n=6 in each group. Values are represented as mean±SEM.

* p<0.05 as compared to diabetic control and

# p<0.05 as compared to glibenclamide (Tukey Kramer multiple comparison test).

**Table 3:** Semi-quantitative evaluation of histological changes in granulation tissue in dead space wound model

**Histological Change**	**Groups**			
	**Diabetic control**	**Glibenclamide**	***Jasminum grandiflorum *** **Linn. flowers**	
**Neutrophils**	1.0±0.36	1.5±0.55	0.9±0.2
**Macrophages**	0.3±0.21	0.8±0.3	1.0±0.0	
**Fibroblasts**	2.5±0.42	1.4±0.2	3±0.36	
**Neo-angiogenesis**	1.8±0.54	1.2±0.16	2.5±0.22	

n=6 in each group. Values are represented as mean±SEM (Dunn multiple comparison test).

## Discussion

This study evaluated wound healing activity of ethanolic extract of *J. grandiflorum *Linn. flowers in diabetic *Wistar *albino rats based on previous observations such as significant anti-lipid peroxidative effect and improvement in the antioxidant defence system (Kolanjiappan and Manoharan, 2005 ▶). Besides, it has been shown that extract of *J. grandiflorum *Linn. flower contains oleacein which has angiotensin converting enzyme (ACE) inhibitory property and ACE inhibitors are patented for their angiogenesis stimulating property (Somanadhan et al., 1998 ▶; Isner, 2001 ▶). *J. grandiflorum *Linn. flowers has been studied for wound healing activity in non-diabetic wounds (Nayak and Krishna, 2007 ▶); however, to the best of our knowledge, the present study is the first one which evaluates wound healing activity of *J. grandiflorum *Linn. flowers in diabetic wounds. 

In the present study, we used a STZ-induced diabetes model as a well-established model to study diabetic wound healing (Sumi et al., 2014 ▶). STZ-induced diabetes model has been shown to exhibit increased superoxide levels (Luo et al., 2004 ▶). Furthermore, it has been demonstrated that in STZ-induced diabetes, angiogenesis is impaired (Teixeira et al., 1999 ▶). Thus, STZ-induced diabetes model provides suitable opportunity to study the effects of a given agent on diabetic wound healing. Glibenclamide was used as positive control as it has been demonstrated that lowering the blood glucose in diabetes helps to promote wound healing (McMurry, 1984 ▶). This positive control group helps to differentiate if ethanolic extract of *J. grandiflorum *Linn. flowers have any additional advantage in promoting diabetic wound healing over blood glucose lowering agents. 

In excision wound group, wound contraction, on the 7^th ^and 14^th ^post-wounding days was significantly more than diabetic control group while on the 11^th^ post-wounding day, it was non-significantly more than diabetic and positive control group. This is in accordance with a previous study which showed significant wound contraction as compared to non-diabetic control (Nayak and Krishna, 2007 ▶). Re-epithelialization on the 11^th^ post-wounding day was higher than diabetic control but could not reach a statistically significant difference. Both of the parameters of excision wound healing i.e. wound contraction and wound re-epithelialization, did not show any significant improvement as compared to glibenclamide which suggests that ethanolic extract of *J. grandiflorum *Linn. flowers has no additional advantage over blood glucose lowering agents. 

Ethanolic extract of *J.grandiflorum* Linn. flowers significantly increased incision wound breaking strength, granulation tissue dry weight and hydroxyproline contents compared to control. Furthermore, *J.grandiflorum *Linn. significantly increased hydroxyproline content in granulation tissue compared to glibenclamide group. These findings are also in accordance with a previous study (Nayak and Krishna, 2007 ▶). Hyperglycemia associated with diabetes mellitus is responsible for the generation of reactive oxygen species (ROS), which in turn create excessive oxidative stress (Schmidt et al., 1994 ▶). Increased oxidative stress leads to activation of matrix metalloproteinases (MMP) which increases collagen degradation and decreases collagen synthesis (Siwik et al., 2001 ▶).The antioxidant property of *J. grandiflorum *Linn. could decrease collagen degradation and increase collagen synthesis which can explain increased dry granulation tissue weight and hydroxyproline content of granulation tissue in treatment group (Umamaheswari et al., 2007 ▶).

Neo-angiogenesis is an important factor in wound healing as it provides necessary elements to the wound bed and is necessary for sustaining the newly formed granulation tissue (Kleinman and Malinda, 2000 ▶). Extracts of *J. grandiflorum *Linn. are found to have oleacein which has ACE inhibitory property (Somanadhan et al., 1998 ▶). The angiogenesis stimulating property of ACE inhibitors is patented with US Patent No. 6,191,144 B1, 2001 (Isner M, 2001 ▶). Thus, oleacein component of *J. grandiflorum *Linn. extract may explain improved neo-angiogenesis in granulation tissue obtained from treatment group.

The methods used for estimation of re-epithelialization in excision wound and hydroxyproline content in granulation tissue, are relatively crude and less sensitive, and hence, limit interpretation of the results. Different doses of extract would be more explanatory; however, we used a single dose in our study, which is a limitation of our study. Furthermore, immunostaining using a blood vessel marker like CD31 could have been more informative for evaluation of angiogenesis in granulation tissue. However, due to limited resources, it was not examined in our setup.

 This study demonstrated that *J. grandiflorum *Linn. significantly enhances wound contraction and granulation tissue formation in diabetic wounds. In addition, it increases new blood vessel formation which results in rapid healing of chronic wounds like diabetic wounds. Thus, it can be useful in accelerating wound healing process in diabetic patients. Further clinical trials in diabetic patients should be done before the clinical application of ethanolic extract of *J. grandiflorum* Linn. flowers.
